# High Efficient Expression and Purification of Human Epidermal Growth Factor in *Arachis Hypogaea* L.

**DOI:** 10.3390/ijms20082045

**Published:** 2019-04-25

**Authors:** Qingshou Yao, Zipeng Yu, Pu Liu, Hao Zheng, Yang Xu, Sixiang Sai, Yuyong Wu, Chengchao Zheng

**Affiliations:** 1 State Key Laboratory of Crop Biology, Shandong Agricultural University, Tai’an 271018, China; yaoqingshou@126.com (Q.Y.); yzp52120090916@163.com (Z.Y.); banbaokezhan@163.com (P.L.); haozheng@genetics.ac.cn (H.Z.); 2 College of Pharmaceutical Sciences, Binzhou Medicial University, Yantai 264003, China; saisixiang@126.com (S.S.); wuyuyong_820@126.com (Y.W.); 3 Shandong Peanut Research Institute, Shandong Academy of Agricultural Sciences, Qingdao 266100, China; xy52120092661@163.com

**Keywords:** *Arachis Hypogaea* L., Hairy roots, Production system, Human epidermal growth factor, *Agrobacterium rhizogenes* R1601

## Abstract

Background: Human epidermal growth factor (hEGF) has drawn intense research attention due to its potential ability to promote healing of serious injuries, such as cuts, burns, and diabetic ulcers. Although hEGF displays prospective clinical value, the growth factor is restricted to the treatment of chronic diabetic ulcers because of its high production cost. Methods: Leguminous plant peanut (*Arachis hypogaea* L.) hairy roots contain relatively few toxic and harmful substances, and tested as an excellent production system for hEGF in our study. To explore the possibility of hEGF expression in peanut, *hEGF* overexpression hairy roots were obtained by infecting leaves with *Agrobacterium rhizogenes* R1601. Results: The maximum transgenic hairy roots inducing rate was 82%. Protein purification and mass spectrometry assays showed that the protein expressed in peanut hairy roots was identified as hEGF. Furthermore, Methylthiazolyldiphenyl-tetrazolium bromide assay showed that hEGF promoted HL-7702 liver cells proliferation, which indicate that hEGF has biological activity and non-toxic on human cells. Conclusion: Our results demonstrate the capacity of peanut hairy root cultures as a controlled, sustainable, and scalable production system that can be induced to produce valued human proteins, such as hEGF.

## 1. Introduction

Human epidermal growth factor (hEGF), a 53-amino acid peptide with three disulfide bonds, promotes the proliferation and differentiation of epithelial cells [1,2,3]. hEGF is specially recognized by membrane-localized receptor, hEGF receptor (EGFR) in human and initiates a series of intracellular signalling cascades, including the synthesis of proteins, the improvement of DNA topoisomerase activity and the activation of key genes involved in cell proliferation, differentiation, and migration [4,5]. Moreover, hEGF has been applied to many disease treatments based on the characteristic of promoting the growth of epidermal cells. However, owing to the high production cost of hEGF, treatment with the growth factor is limited to chronic diabetic ulcers.

The mass production of hEGF mainly contains three most common methods. Firstly, the isolation of hEGF from batches of fresh human urine, blood, breast milk, and gastric juice has been largely reported [6]. However, endogenous concentration of hEGF is very low (50 ng/mL), and most of hEGF forms complex with receptor EGFR in vivo. The isolation and purification of hEGF in gram quantities has become a formidable task [6]. In addition, hEGF with three disulfide bridges cannot be produced as soluble, active, and correctly folded protein via chemical synthesis method. Thirdly, producing heterologous proteins like hEGF from prokaryotic and eukaryotic organisms in large quantities based on recombinant DNA technology placed increased impetus on the development of more effective and economical methods for industrial purposes [7].

hEGF has been expressed in various prokaryotic and eukaryotic systems, including *Escherichia coli*, yeast, rice, and tobacco [8,9,10,11]. Furthermore, the bioactive hEGF has been reported to be highly expressed in tobacco hairy roots, thus demonstrating the feasibility of expressing hEGF protein in the hairy root production system [12]. As production systems, hairy root cultures have a number of advantages, such as rapid growth rate, high productivity, genetic/biochemical stability, and hormone-autotrophy [13]. Therefore, the hairy root systems have been widely used to produce recombinant pharmaceutical proteins, such as human acetylcholinesterase, interleukin 12, and human tissue-plasminogen activator [14,15,16]. The application of hairy root cultures to the pharmaceutical industry is believed to be of great potential because of its high efficiency and relatively low cost [17].

As an excellent feed used in animal husbandry, peanut contains no hazardous substances and, thus, may be used to isolate and purify hEGF safely. In the previous studies, Parsons described the properties of the peanut hairy root system, and concluded that this system offered new possibilities for expressing exogenous proteins [12]. To construct the expression system of *hEGF* in peanut hairy roots, *hEGF* was inserted into pCAMBIA1300 vector, and the recombined vector was transformed into *Agrobacterium rhizogenes* R1601 to obtain the engineering strain (designated as HR1601). Peanut leaves were infected by HR1601, and the *hEGF*-overexpressing (OE) hairy root lines were induced and developed at the inoculation site. The protein purification and mass spectrometry assays showed that the protein expressed in peanut hairy roots was target protein hEGF. Moreover, the hEGF was demonstrated to be bioactive and non-toxic on human cells. Taken together, this study gives strong evidence that the peanut hairy roots system is suitable for the production of hEGF protein.

## 2. Results

### 2.1. The Establishment of Protein Expression System in Peanut Hairy Root

**Figure 1:**
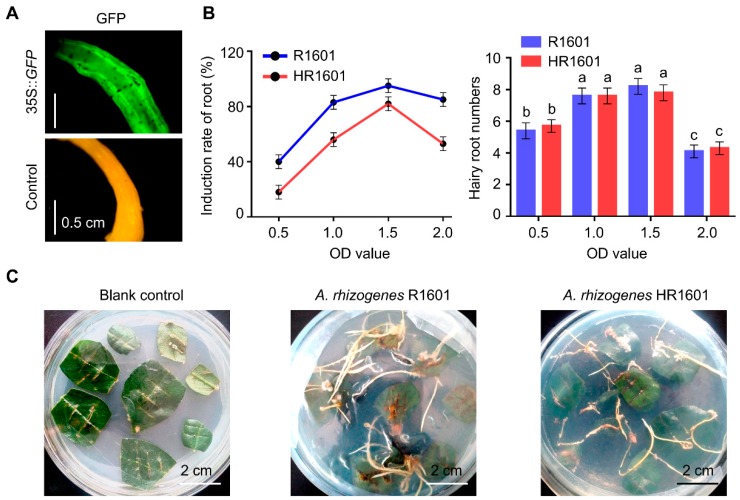
The establishment of peanut hairy root protein expression system. (**A**) Green fluorescence Pof the peanut hairy roots detected by fluorescent inverted microscopy. 35S::*GFP*, peanut hairy roots developed from leaves infected with the recombined GFP construct. Control, peanut hairy roots developed from leaves infected with *Agrobacterium rhizogenes* R1601. (**B**) The induction rate and total numbers of peanut hairy roots inoculated with various concentrations of R1601 and HR1601 (*Agrobacterium rhizogenes* R1601 transformed with human epidermal growth factor (hEGF)). The best induction rate is obtained when its OD_600_ value reached 1.5 both for R1601 and HR1601. Error bars indicate SEM (*n* = 3), *p* < 0.05. One-way ANOVA Duncan’s test is used for statistical analysis. Statistical differences are indicated by lowercase letters and different letters represent different significance. This experiment was repeated three times with similar results. (**C**) Peanut leaves infected with *Agrobacterium rhizogenes* R1601 or HR1601 with hairy roots formation. Blank control, non-infected peanut leaves with no hairy roots formation.

To establish the protein expression system in peanut hairy root, the green fluorescent protein (*GFP*) gene was constructed into the pCAMBIA1300 expression vector, which was used as the experimental vector to explore and optimize the induction conditions of peanut hairy roots. As shown in Figure 1A, fluorescence of GFP was clearly observed in peanut roots inoculated with *Agrobacterium rhizogenes* R1601 containing expression vector 35S::*GFP*, which indicated the feasibility of this system. The details description of this method was listed in “Materials and Methods”. Subsequently, the infection solution containing different concentrations of *Agrobacterium rhizogenes* was used to test the root induction efficiency, and the results showed that the optimal concentration of root induction was OD_600_ = 1.5 (Figure 1B).

To ligate the *hEGF* gene in pCAMBIA1300 vector, *Bam*HI and *Sac*I restriction sites were embedded at the forward primer and reverse primer, respectively. The plasmid was subsequently transformed into *Agrobacterium rhizogenes* R1601, and the strain carrying *hEGF* was designated as HR1601. The sterilized peanut leaves were soaked in the infection solution containing R1601 or HR1601 for 2 min, and then transferred to MS solid medium to induce hairy roots. When growing to the third week, multiple root tips developed at the inoculation sites of the leaves, and after another two weeks, the root tips grew into real hairy roots about 2–4 cm length (Figure 1C). These results comprehensively proved that the protein expression system of peanut hairy roots was successfully established, and the *hEGF* gene may also be successfully induced in the generation of hairy roots.

### 2.2. The Identification of the hEGF Transgenic Hairy Roots

**Figure 2:**
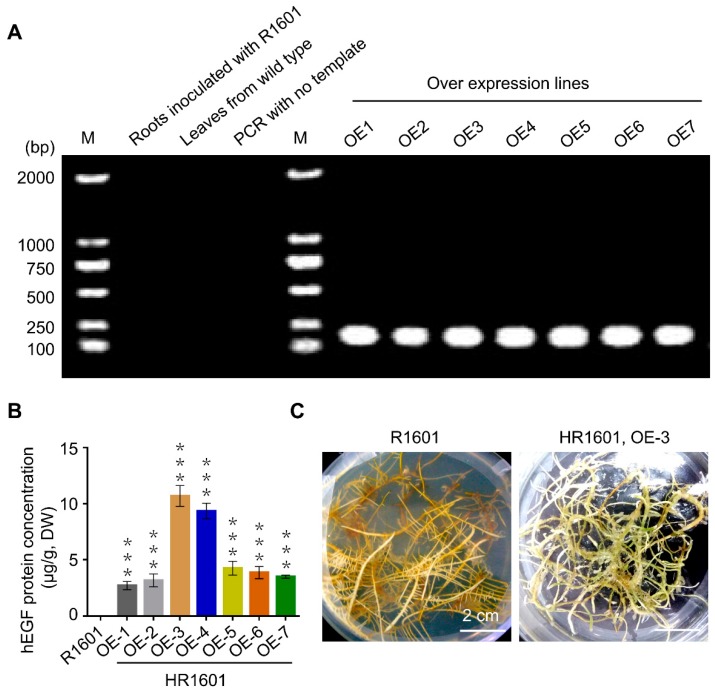
The identification of the *hEGF* transgenic hairy roots (**A**) PCR-based analysis of the recombined *hEGF* construct in various peanut hairy root lines. Lane 2, PCR amplification uses the template of hairy roots inoculated with *Agrobacterium rhizogenes* R1601; Lane 3, PCR amplification uses the template of native peanut leaves; Lane 4, negative control with no template; Lanes 6–12, PCR amplification uses the template of different peanut hairy root lines infected with *Agrobacterium rhizogenes* HR1601. M, marker. (**B**) Protein expression analysis of the hEGF protein in various peanut hairy root lines. Total proteins of transgenic hairy roots and control (hairy roots infected with *Agrobacterium rhizogenes* R1601) were examined by a special ELISA assay, in which hEGF peptide was designed as the special antigen. Error bars indicate SEM (*n* = 3). *** *p* < 0.001 (Student’s *t*-test). These experiments were repeated three times with similar results. (**C**) Peanut hairy roots developed from leaves infected with *Agrobacterium rhizogenes* R1601 or HR1601. No callus are found on the surface of hairy roots infected with R1601 after two weeks in MS medium, and the new hairy roots are smooth. While white callus are found on the surface of hairy roots infected with HR1601.

To identify the positive transformed roots of *hEGF*, PCR assay was performed using the DNA extracted from hairy roots inoculated with R1601 or HR1601 as the template. Seven possibly *hEGF* overexpression lines were obtained, which demonstrated that the *hEGF* gene was successfully transformed into hairy roots (Figure 2A). To confirm whether the *hEGF* gene integrated into the peanut genome and had the ability for expressing the target protein, a special ELISA assay was performed, which hEGF peptide was designed as the special antigen. As shown in Figure 2B, hEGF was successfully expressed in all overexpression lines, and the protein concentration of hEGF was highest in OE-3 line.

Interestingly, the hairy roots transformed with HR1601 showed slower growth rates compared with those infected with R1601 after two weeks of culture (Figure 2C). Cells with hairy roots infected with R1601 showed a rapid growth rate and the phenotype of hairy roots look white and smooth (Figure 2C). This result may be explained by the fact that the continuous high expression of exogenous hEGF peptide restricts the expression of other proteins in peanuts to some extent.

### 2.3. The Isolation and Purification of hEGF Peptide in Hairy Roots

**Figure 3:**
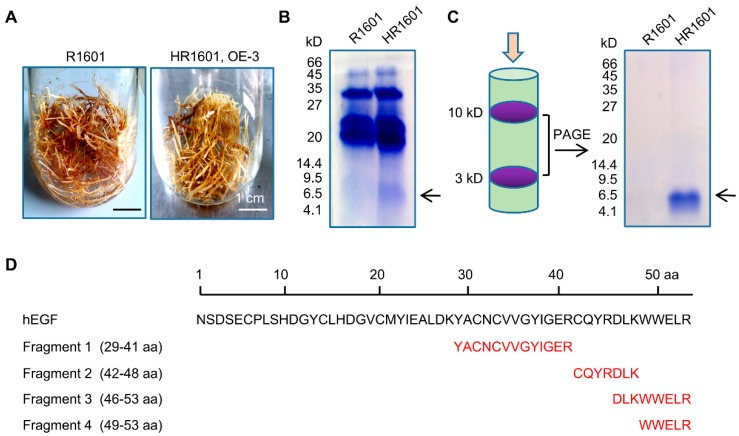
The identification of hEGF via matrix-assisted laser desorption/ionization time of flight mass spectrometry (MALDI-TOF-MS). (**A**) Pictures represent the peanut hairy root lines cultured in 100 mL of MS liquid medium for one month. Peanut hairy roots infected with *Agrobacterium rhizogenes* R1601 were used as the negative control. (**B**) SDS-PAGE analysis of the total protein samples extracted from peanut hairy roots infected with *Agrobacterium rhizogenes* R1601 or HR1601. Protein band indicated by the arrow is hEGF protein. (**C**) SDS-PAGE was used to isolate hEGF protein from the interception proteins again. The target protein hEGF is about 6 kDa. Protein band indicated by the arrow is hEGF protein. (**D**) Proteolytic enzyme trypsin was used to perform specific protein digestion at lysine and arginine and four short peptide sequence were identified by MALDI-TOF-MS analysis.

To further confirm the protein expressed by hairy roots was hEGF, hairy roots of OE-3 were cultured in large scale in 100 mL of MS liquid medium for total protein extraction, and the roots infected with R1601 were used as the negative control (Figure 3A). As shown in Figure 3B, a band near 6.5 kDa (hEGF ≈ 6 kDa) could be clearly observed in the SDS-PAGE gel. Subsequently, the total amount of protein was filtered with a Millipore super filter, and the proteins between 3 kDa and 10 kDa were retained. Then, the proteins between 3–10 kDa were separated by SDS-PAGE again to isolate the hEGF protein and remove impurities as much as possible (Figure 3C). It can be seen that the target protein was well separated, and the protein bond approximately 6 kDa was subsequently cut and identified by matrix-assisted laser desorption/ionization time of flight mass spectrometry (MALDI-TOF-MS) analysis (Supplementary Figure S1). Blast analysis showed that the four identified peptides could match the protein sequence of hEGF and cover a total of 25 amino acid sequences from the 29th to the 53th, with a total protein coverage of 47%, which gave the strong evidence that the protein expressed and purified in peanut hairy roots was hEGF (Figure 3D).

### 2.4. The hEGF Protein Purified from Peanut Hairy Roots Has Biological Activity

**Figure 4:**
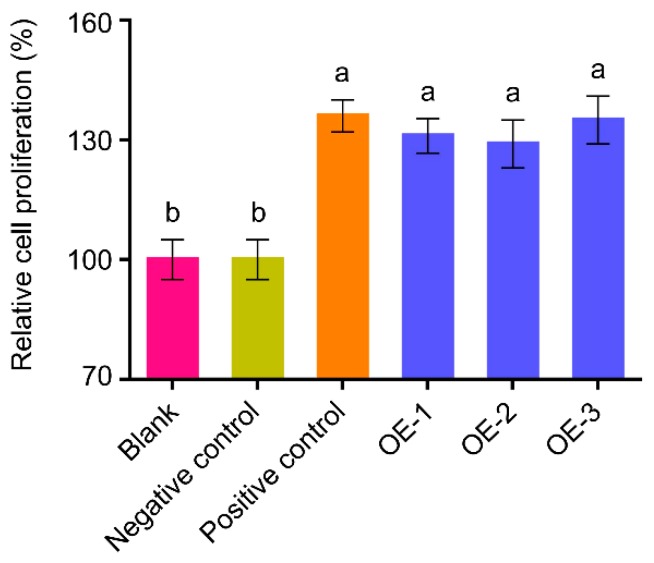
The hEGF protein purified in peanut hairy roots has biological activity. Biological activity of hEGF on human liver cells. Blank, the normal human liver cell HL-7702 cells; Negative control, the interception proteins from hairy roots infected with R1601 are supplemented with HL-7702 cells; Positive control, the hEGF peptide obtained from Sangon (Shanghai, China); OE-1, OE-2 and OE-3, the interception proteins from three independent hEGF transgenic peanut hairy root lines are each supplemented with HL-7702 cells. Error bars indicate SEM (*n* = 3), *p* < 0.01. One-way ANOVA Duncan’s test is used for statistical analysis. Statistical differences are indicated by lowercase letters and different letters represent different significance. These experiments were repeated three times with similar results.

Biological activity of the hEGF expressed in peanut hairy roots was detected to assess its effectiveness in promoting human liver cell HL-7702 cells proliferation. Interception protein mixture obtained from different hairy roots was added to HL-7702 cells culture system. As shown in Figure 4, the interception protein mixture obtained from OE-1, OE-2 and OE-3 significantly stimulated the cell proliferation and enhanced cell viability, while no significantly differences were observed between interception protein mixture obtained from roots inoculated with R1601 and the control with no treatment, indicating that hEGF, rather than other proteins or growth factors, promoted cells proliferation. Taken together, peanut hairy root system is suitable for the production of hEGF protein, and the hEGF is bioactive and non-toxic on human cells.

## 3. Discussion

hEGF has been widely reported to promote proliferation of a wide range of cells, such as epithelial cells, endothelial cells and fibroblast cells, which can be used as a perfect cut healing agent for various wound healing and skin regeneration [18]. Therefore, the clinical application value or market demand of hEGF has become huge and booming in recent years. Hairy root is a good host for mass production of recombinant protein due to its abundant quality and extensive lateral branches [19]. Hairy root production system has received considerable interest in recent years [20,21]. As promising systems for protein production, hairy root cultures have been used to obtain vaccine, antibody, and multiple clinical proteins [22,23]. As an important food crop with low potential risk, peanut hairy root system is consistently capable of producing recombinant proteins in a more native, massive and lower risk pattern than those of other plant production system [11,14,24]. This study successfully expressed and purified hEGF in peanut hairy roots, and the maximum hEGF protein content in OE-3 line was 10.7 µg/g, indicating that peanut hairy root system is efficient to produce this commercial protein. Taken together, peanut hairy roots may provide a novel potential platform for the production of a variety of human or animal proteins, at least hEGF.

Notably, the growth rate of overexpressed hEGF hairy roots was lower than that of the normal control group (Figure 2C and Figure 3A), which can be explained by the fact that the continuous high expression of exogenous hEGF protein may restrict the expression of other proteins in peanuts in some unknown mechanism. As the positive regulator for cell growth in human, hEGF lacks the specific receptor(s) in peanut hairy roots, thus it cannot exert the role in promoting cell growth in peanut root system.

Peanuts as one of the five most important oilseed crops, serves as a good source of proteins, calories, vitamins and minerals. Thus, these food crops have low potential risk to contain hazardous substances for people and animals. Previous study exhibited that opines, resveratrol and stilbene derivatives were detected in peanut hairy roots [20]. In our study, resveratrol contents in different transgenic lines were examined and the results showed that transgenic lines OE-1, OE-2, OE-3 and OE-4 had very similar contents of resveratrol (Supplementary Figure S2), while the protein levels of hEGF were diverse in these transgenic lines (Figure 2B). Therefore, our study indicates that resveratrol does not interfere with the expression of hEGF in peanuts. In addition, the molecular sieve purification system can eliminate most of hazardous substances and obtain the highly purified hEGF ultimately.

Conversion of the hEGF protein into extracellular protein instead of cytoplasmic protein by modification of the expression construct and sub-cellular targeting will be attempted in future work. Furthermore, optimizing the culture medium or other measures in appropriate method may remarkably improve the expression levels of hEGF in peanut hairy root system. Taken together, all these results demonstrate the capacity of peanut hairy root cultures as a controlled, sustainable and scalable production system that can be induced to produce valued human proteins, like hEGF.

Plant hairy root system has been favored by researchers in recent years and various studies have emerged implicating hairy root system to be widely applied in clinical field. Clinical safety and protein expression ability are two pivotal problems in clinical field. *Brassica rapa* hairy root has been established in 2019, and human alpha-L-iduronidase can undergo the removal of N-terminal signal peptide and *O*-glycosylation modification in the expression system, thus demonstrating the feasibility of expressing heterologous protein in the hairy root production system [23]. In addition, hEGF produced in our study can promote human liver cells (HL-7702 cells) grown via cell proliferation assay (Figure 4), suggesting that the hEGF produced in peanut hairy root system has biological activity. Thus, we make an assumption that the bioactive hEGF may also undergo correct post-translational modification process, including the formation of intramolecular disulfide bonds and *O*-fucosylation modification process in peanuts.

*Brassica rapa* and peanuts as food crops are good hosts for mass production of recombinant protein. The activated hEGF has been reported to be highly expressed in tobacco hairy roots (2 μg/g, fresh weight) [12], which is slightly higher than the peanut hairy roots system (10.7 μg/g, dry weight) in our study. However, harmful substances such as nicotine in the tobacco system has restricted the wide application of tobacco hairy root system in the clinical field. The development of molecular sieve purification system can lay a solid foundation for further exploitation of the system in the future.

## 4. Materials and Methods

### 4.1. Plant Material and Bacteria Strains

The pCAMBIA1300 expression vector used in this research was stored in Chengchao Zheng’s laboratory at the State Key Laboratory of Crop Biology, Shandong Agricultural University (Tai-an, Shandong, China). The peanut seeds (Luhua 11), *Escherichia coli* DH5α, and *Agrobacterium rhizogenes* R1601 were stored at the Gene Engineering Laboratory of the College of Pharmacy, Binzhou Medical University (Yan-tai, Shandong, China). The peanut seeds were stored at room temperature after drying and reproduced once a year. The strains of *Escherichia coli* DH5α and *Agrobacterium rhizogenes* R1601 were stored at −80 °C and activated once a year.

### 4.2. The Construction of HR1601 Strain

The *hEGF* gene sequence was 165 base pair (bp) long and synthesized by Sangon (Shanghai, China) with the reference to the sequence demonstrated by Yamagata in 1989 [25]. The primers for specific amplification of *hEGF* were designed as follows: forward primer, GGA TCC ATG AAC AGT GAT TCA GAA TGT CCT C (*Bam*HI) and reverse primer, GAG CTC CTA TCG CAG TTC CCA CCA TTT C (*Sal*I). The PCR product was sub-cloned into pCAMBIA1300 expression vector with recognition sites for *Bam*HI and *Sal*I. Then, the expression vector was transformed into *Agrobacterium rhizogenes* R1601 competent cells, and the *Agrobacterium rhizogenes* R1601 with expression vector was designated as HR1601. The restriction enzymes, *Bam*HI and *Sac*I, were purchased from Takara Biotech (Dalian, China). DNA polymerase (2 × Phanta Max Master Mix, p515) was purchased from Vazyme (Nanjing, China). Trans DNA Marker and EasyPure^®^ Quick Gel Extraction Kit were purchased from TransGen (Beijing, China).

### 4.3. The Establishment of Peanut Hairy Root Culture System

Seeds of peanuts were germinated on soil and grown under greenhouse conditions (25 °C and approximately 16 h light /8 h dark photoperiod) for 30 d. Surface-sterilized leaf disks (approximately 2 × 2 cm) were inoculated with various concentrations of *Agrobacterium rhizogenes* R1601 and HR1601 strain (OD_600_ = 0.5, 1.0, 1.5 or 2.0) for 2 min [19]. After removal of the excess strain with distilled water, leaf disks were then transferred to Murashige and Skoog medium (MS) containing cefotaxime sodium (200 mg/L) and allowed to grow in continuous light (8800 LUX) at 25 °C for about three weeks for hairy root emergence. The materials were subsequently cultured at 25 °C (16 h light/8 h dark, 8800 LUX) for two weeks and the roots were about 2 cm length. The root elicitation rate was calculated with the following formula: the number of leaves with initiated roots/the number of leaves infected by R1601 or HR1601. Hairy roots were cut away from the leaf blade base and transferred to MS medium to culture for another two weeks, and the roots were about 6–10 cm length. Roots of maturation zone (about 1 cm) were used to extract DNA to screen transgenic hairy roots. Then, transgenic hairy roots were transferred to 100 mL of MS liquid medium to culture in large scale for another one month for the protein extraction. In this study, 35S::*GFP* (pBI121-GFP expression vector) has been used in advance to test the effectiveness of this system, and the fluorescence of GFP was monitored by fluorescent inverted microscopy (Olympus, IX71, Tokyo, Japan). At least 20 individual roots were analyzed.

### 4.4. Hairy Root Growth and hEGF Protein Content Analyses

Seven *hEGF* overexpression lines were sub-cultured for one month at 25 °C in 100 mL of MS medium, with each line separated into three parallel samples, and the peanut hairy roots infected by R1601 were used as the negative control. After removal of the excess MS medium with distilled water, the root samples were used for protein concentration analysis. The total proteins of hairy roots (0.1 g, dry weight) were extracted using the Plant Total Protein Extraction Kit (Sangon, Shanghai, China). After dissolution in 10 mM phosphate buffer (Solarbio, Beijing, China), the hEGF protein concentrations were measured by ELISA kit (Sbjbio, Nanjing, China) according to the manufacturer’s instructions.

### 4.5. The Detection of the hEGF Protein by Matrix-Assisted Laser Desorption/Ionization Time of Flight Mass Spectrometry

The total protein extracted by Kit were filtered with Millipore Super filter (Millipore, Billerica, MA, USA), and the proteins between 3 kDa and 10 kDa were retained. The SDS-PAGE electrophoresis was subsequently performed to isolate the hEGF protein, and the protein bond approximately 6 kDa was identified by matrix-assisted laser desorption/ionization time of flight mass spectrometry (MALDI-TOF-MS) (Sangon, Shanghai, China). Then hEGF was digested into short peptides by trysin that can catalyze the hydrolysis at the specific amino acids (lysine or arginine). MALDI-TOF-MS analysis showed that four sequenced short peptides could match the protein sequence of hEGF via sequence alignment analysis, which match 47% of the total protein sequence of hEGF (29–53 amino acid). The protein sequence of hEGF was obtained and downloaded from (https://www.ncbi.nlm.nih.gov/nucleotide/341526?Report=genbank&log$=nuclalign&blast_rank=1&RID=DVGPY9WK016).

### 4.6. Digestion Before MS Analysis

In gel digestion: according to Katayama et al. [26]. (1) Wash the gel spot twice. (2) Remove the water and de-stained the gel spot at 23 °C for 30 min. (3) Remove the de-stained solution and add dehydration solution1 for 30 min. (4) Remove the dehydration solution1 and add dehydration solution2 for 30 min. (5) Remove the dehydration solution2 and add reduction solution1 at 57 °C for 1 h. (6) Remove the reduction solution1 and add reduction solution2 at 23 °C for 30 min. (7) Remove the reduction solution2 and add imbibition solution at 23 °C for 10 min. (8) Remove the imbibition solution and add dehydration solution1 for 30 min. (9) Remove the dehydration solution1 and add dehydration solution2 for 30 min. (10) The gels were rehydrated in 10 µl digest solution for 30 min. (11) 20 µL cover solution was added for digestion 16 h at 37 °C. (12) The supernatants were transferred into another tube. (13) The gels were extracted once with 50 µL extraction buffer at 37 °C for 30 min. The peptide extracts and the supernatant of the gel spot were combined and then completely dried.

### 4.7. MS Analysis

(1) Samples were re-suspended with Nano-RPLC buffer A. (2) The online Nano-RPLC was employed on the Eksigent nanoLC-Ultra™ 2D System (AB SCIEX). The samples were loaded on C18 nanoLC trap column (100 µm × 3 cm, C18, 3 µm, 150 Å) and washed by Nano-RPLC Buffer A (0.1% FA, 2% ACN) at 2 μL/min for 10 min. (3) An elution gradient of 5–35% acetonitrile (0.1% formic acid) in 30 min gradient was used on an analytical ChromXP C18 column (75 μm × 15 cm, C18, 3 μm, 120 Å) with spray tip. (4) Data acquisition was performed with a Triple TOF 5600 System (AB SCIEX, USA) fitted with a Nanospray III source (AB SCIEX, Foster city, CA, USA) and a pulled quartz tip as the emitter. Data were acquired using an ion spray voltage of 2.5 kV, curtain gas of 30 PSI, nebulizer gas of 5 PSI, and an interface heater temperature of 150 °C. For information dependent acquisition (IDA), survey scans were acquired in 250 ms and as many as 35 product ion scans were collected if they exceeded a threshold of 150 counts per second (counts/s) with a 2+ to 5+ charge-state. The total cycle time was fixed to 2.5 s. A rolling collision energy setting was applied to all precursor ions for collision-induced dissociation (CID). Dynamic exclusion was set for ½ of peak width (18 s). And the precursor was then refreshed off the exclusion list. (5) Based on combined MS and MS/MS spectra, proteins were successfully identified based on 95% or higher confidence interval of their scores in the MASCOT V2.3 search engine (Matrix Science Ltd., London, UK.), using the following search parameters: Uniprot-Komagataella pastoris (Strain ATCC 28485) database. Trypsin as the digestion enzyme. Two missed cleavage site. Fixed modifications of Carbamidomethyl (C). Partial modifications of Acetyl (Protein N-term), Deamidated (NQ), Dioxidation (W), Oxidation (M). ± 10 ppm for precursor ion tolerance and ± 0.6 Da for fragment ion tolerance.

### 4.8. Cell Proliferation Assay

The biological activity of hEGF produced by transgenic hairy roots was analyzed by human liver cell HL-7702 cell proliferation assay described by Pan [27]. Same quality of hairy roots (1 g, fresh weight) infected with R1601 or HR1601 (OE1, OE2 and OE3) were used to extract the total proteins. Vacuum freeze drying the total proteins, and dissolved in sterile water, and then filtered with Millipore Super filter. The proteins between 3 kDa and 10 kDa were retained. Methylthiazolyldiphenyl-tetrazolium bromide (MTT) assay was performed to test the bioactivity of hEGF via promoting proliferation of human liver cells (HL-7702 cells) grown in medium RPMI-1640 supplemented with 10% fetal bovine serum on sterile 96-well tissue culture plate at 37 °C and 5% CO_2_ for 24 h. HL-7702 cells were supplemented with 10 μL hEGF (0.1 ng/μL) expressed in the hairy roots and incubated for 24 h. Then 20 μL MTT (5 mg/mL) solutions/well were added to cells and the plates were incubated for additional 4 h. The crystals were then dissolved in 100 μL dimethyl sulfoxide per well. Absorbance was recorded at a wavelength of 490 nm with a microtiter plate reader (Bio-Tek ELX800, Winooski, VT, USA). Human liver cells HL-7702 were obtained from the Type Culture Collection of the Chinese Academy of Sciences (Shanghai, China).

### 4.9. Statistical Tests

All experiments were performed at least three independent repetitions (*n* ≥ 3). One-way ANOVA Duncan’s test (*p* < 0.05 for Figure 2B; *p* < 0.01 for Figure 4) and Student’s *t*-test (*** *p* < 0.001) in Statistical Product and Service Solutions 24 (SPSS 24, IBM, Armonk, NY, USA) were used for statistical analysis.

## 5. Conclusions

We successfully expressed hEGF protein with high clinical value in the hairy root system of peanut, and confirmed the biological activity of hEGF through cell proliferation experiments. In summary, this study provides an important reference for the mass production of hEGF and an important feasible idea for the substantial cost reduction of hEGF.

## Supplementary Materials

Supplementary materials can be found at https://www.mdpi.com/1422-0067/20/8/2045/s1. Figure S1. Identification of hEGF protein via MALDI-TOF-MS. Figure S2. The contents of resveratrol in different hEGF transgenic hairy roots.

## Abbreviations

hEGF	Human epidermal growth factor
EGFR	hEGF receptor
MTT	Methylthiazolyldiphenyl-tetrazolium bromide
OE	overexpression lines
bp	base pair
MS	Murashige and Skoog medium
MALDI-TOF-MS	matrix-assisted laser desorption/ionization time of flight mass spectrometry
