# Supplementary material for: High Efficient Expression and Purification of Human Epidermal Growth Factor in Arachis Hypogaea L
Source: Int J Mol Sci. 2019 Apr 25;20(8):2045. doi: 10.3390/ijms20082045 (PMC6515032; doi:10.3390/ijms20082045)
Supplement: Supplementary file 1 [file ijms-20-02045-s001.pdf]

Supplementary Figure S1

Mass spectrometry

Protein View

Match to: **201809994** Score: **2257**

Found in search of C:\Users\DELL\Desktop\LCMSMS\10.22LCMCMS\09994-2.mgf

Nominal mass (M_r): **6691**; Calculated pI value: **4.78**
NCBI BLAST search of 201809994 against nr
Unformatted sequence string for pasting into other applications

Fixed modifications: Carbamidomethyl (C)
Variable modifications: Acetyl (Protein N-term),Deamidated (NQ),Dioxidation (W),Oxidation (M)
Cleavage by Trypsin: cuts C-term side of KR unless next residue is P
Sequence Coverage: **46%**

Matched peptides shown in **Bold Red**

```
 1 MNSDSECPLS HDGYCLHDGV CMYIEALDKY ACNCVVGYIG ERCQYRDLKW
51 WELR
```

Start - End	Observed	Mr(expt)	Mr(calc)	ppm	Miss	Sequence	
30 - 42	780.8501	1559.6856	1559.6861	-0	0	K.YACNCVVGYIGER.C	(Ions score 71)
43 - 49	491.7436	981.4727	981.4702	3	1	R.CQYRDLK.W	(Ions score 4)
47 - 54	573.3063	1144.5981	1144.6029	-4	1	R.DLKWWELR.-	(Ions score 40)
50 - 54	395.2063	788.3980	788.3969	1	0	K.WWELR.-	(Ions score 14)

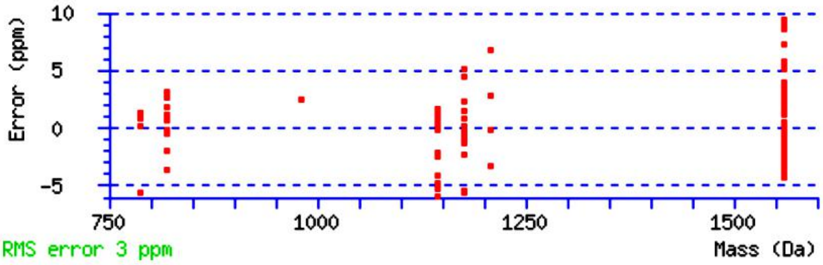

Supplementary Figure S1. Identification of hEGF protein via MALDI-TOF-MS

Proteolytic enzyme trypsin was used to perform specific protein digestion at lysine and arginine and four short peptide sequence were identified by MALDI-TOF-MS analysis.

Supplementary Figure S2

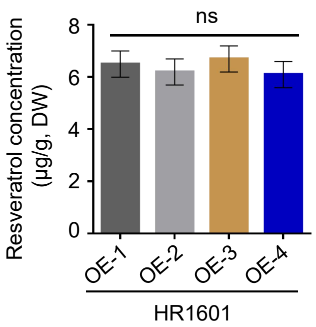

Supplementary Figure S2. The contents identification of resveratrol in different hEGF transgenic hairy roots

Expression analysis of resveratrol in various peanut hairy root lines. Error bars indicate SEM (*n* = 3). Student's *t*-test. ns, no statistic significance. These experiments were repeated three times with similar results. DW, dry weight.
